# Comparison of programmatic data from antenatal clinics with population-based HIV prevalence estimates in the era of universal test and treat in western Kenya

**DOI:** 10.1371/journal.pone.0287626

**Published:** 2023-06-26

**Authors:** Julie Ambia, Julio E. Romero-Prieto, Daniel Kwaro, Kathryn Risher, Sammy Khagayi, Clara Calvert, David Obor, Malebogo Tlhajoane, Fredrick Odongo, Milly Marston, Emma Slaymaker, Brian Rice, Chodziwadziwa Whiteson Kabudula, Jeffrey W. Eaton, Georges Reniers

**Affiliations:** 1 Department of Population Health, London School of Hygiene & Tropical Medicine, London, United Kingdom; 2 Kenya Medical Research Institute - Center for Global Health Research, Kisumu, Kenya; 3 MRC Centre for Global Infectious Disease Analysis, School of Public Health, Imperial College London, London, United Kingdom; 4 Usher Institute, University of Edinburgh, Edinburgh, United Kingdom; 5 MeSH Consortium, Department of Public Health Environments and Society, Faculty of Public Health and Policy, London School of Hygiene and Tropical Medicine, London, United Kingdom; 6 MRC/Wits Rural Public Health and Health Transitions Research Unit (Agincourt), School of Public Health, Faculty of Health Sciences, University of the Witwatersrand, Johannesburg, South Africa; South African Medical Research Council (SAMRC) / Stellenbosch University (SU), SOUTH AFRICA

## Abstract

**Objective:**

To compare HIV prevalence estimates from routine programme data in antenatal care (ANC) clinics in western Kenya with HIV prevalence estimates in a general population sample in the era of universal test and treat (UTT).

**Methods:**

The study was conducted in the area covered by the Siaya Health Demographic Surveillance System (Siaya HDSS) in western Kenya and used data from ANC clinics and the general population. ANC data (n = 1,724) were collected in 2018 from 13 clinics located within the HDSS. The general population was a random sample of women of reproductive age (15–49) who reside in the Siaya HDSS and participated in an HIV sero-prevalence survey in 2018 (n = 2,019). Total and age-specific HIV prevalence estimates were produced from both datasets and demographic decomposition methods were used to quantify the contribution of the differences in age distributions and age-specific HIV prevalence to the total HIV prevalence estimates.

**Results:**

Total HIV prevalence was 18.0% (95% CI 16.3–19.9%) in the ANC population compared with 18.4% (95% CI 16.8–20.2%) in the general population sample. At most ages, HIV prevalence was higher in the ANC population than in the general population. The age distribution of the ANC population was younger than that of the general population, and because HIV prevalence increases with age, this reduced the total HIV prevalence among ANC attendees relative to prevalence standardised to the general population age distribution.

**Conclusion:**

In the era of UTT, total HIV prevalence among ANC attendees and the general population were comparable, but age-specific HIV prevalence was higher in the ANC population in most age groups. The expansion of treatment may have led to changes in both the fertility of women living with HIV and their use of ANC services, and our results lend support to the assertion that the relationship between ANC and general population HIV prevalence estimates are highly dynamic.

## Background

Estimates of levels and trends in HIV prevalence within high-burden countries in sub-Saharan Africa (SSA) are extrapolated from HIV prevalence trends from antenatal care (ANC) clinic populations to the general population [1, 2]. In the context of a generalised HIV epidemic, where transmission is predominantly heterosexual, HIV testing amongst pregnant women has provided essential data for HIV surveillance, both locally and globally [3]. While several limitations have been identified [4], these data have been critical in helping policy makers to design appropriate HIV prevention and treatment programmes; to monitor testing and treatment coverage, and to distribute resources efficiently. In addition, high quality ANC data constitute key inputs for mathematical models of HIV epidemics at national and sub-national levels [5, 6].

Early studies comparing ANC prevalence estimates to population-based surveys reported prevalence to be lower in the general population [7–10], resulting in a recommended downward adjustment of ANC estimates by a factor of 0.2 when extrapolating to the general population [11]. ANC-based estimates of age-specific HIV prevalence estimates were characterised by an upward bias amongst younger age groups, and a downward bias amongst older women [12]. Further analyses suggested that at younger ages these differences were largely driven by a selection effect, where HIV infection among pregnant and sexually active women was much more common than among their non-pregnant peers, many of whom had never had sex [13]. Conversely, at older ages, HIV-associated sub-fertility led to the underrepresentation of women living with HIV in the ANC data [14].

Following the rollout of antiretroviral therapy (ART), fertility rates of women living with HIV have increased, though these remain lower than fertility rates for HIV-negative women [15, 16]. The expansion of ART services has led to a reduction in HIV-related mortality and an associated shift in the age profile of women living with HIV, a reduction in the prevalence of widowhood, and changed fertility intentions amongst women living with HIV [15, 17]. ‘Option B+’ for prevention of mother-to-child HIV transmission was introduced to provide ART to all pregnant women living with HIV irrespective of their CD4-count in 2012 [18]. This was further expanded in 2016 as the World Health Organization recommended ART for all persons diagnosed with HIV, regardless of their clinical stage of infection or CD4 count following the approach known as universal test-and-treat (UTT) [19]. As such, it is important to investigate the extent to which ANC programmatic data represents HIV prevalence estimates in the general population in the era of UTT.

This paper compares the total and age-specific HIV prevalence in pregnant women attending ANC clinics and the general population of women aged between 15 and 49 years living within a demographic surveillance area in Siaya County in western Kenya in the era of UTT. Decomposition was conducted to assess the contributions of different age groups towards the differences in HIV prevalence estimates observed in both populations.

## Methods

### Study population

Data for this analysis were collected from Siaya County in Western Kenya. In 2017, Siaya County had an HIV prevalence of 21% among adults aged 15–49 years, the highest in the country. Women in Siaya County were four times more likely to be infected with HIV (22.4%) than all women nationwide (5.2%) [20]. The majority of the women in this county report having been tested for HIV [21], and about 95% of the women attended at least one ANC visit during pregnancy, where they receive provider-initiated HIV testing and counselling [22]. In 2014, women in Siaya County had an average of 4.2 lifetime births [23].

A Health and Demographic Surveillance System (HDSS) was established in Siaya County in 2001 by the Kenya Medical Research Institute (KEMRI) in collaboration with the United States Centers for Disease Control and Prevention (CDC) [24]. The Siaya HDSS is divided into three areas, Asembo, Gem, and Karemo. This analysis was restricted to Gem in which the HDSS conducts HIV sero-surveys. Within Gem, there are 142 villages and 13 healthcare facilities that serve patients who primarily reside in this rural area.

The study population for this analysis comprised of two groups. Firstly, the ANC population were pregnant women, aged between 15 and 49 years, residing in Gem and who visited any of the 13 ANC clinics in 2018. Secondly, the general population comprised of a population-based random sample of 15–49 year old women residing in the Gem area in 2018.

### General population HIV sero-survey

The general population sample was randomly selected from a sampling frame of 15,000 compounds in the Gem HDSS area in October 2010. A two-step random sampling approach was used to select compounds. One of the 25 community leaders picked a paper from a bucket with a unique registration number of a compound followed by the study statistician who picked a computer-generated random number until 50% of all compounds were drawn [25]. A study population of 7,000 compounds were randomly selected and 39,680 individuals participated in the HIV sero-survey [26].

Within these compounds, HDSS residents and non-residents aged above 13 years were invited to test for HIV. This sample included individuals who had spent the previous night in one of the households and consented for HIV testing [26]. Siaya HDSS revisited this open cohort of individuals every 12 to 24 months. However, due to changes based on the composition of households, out-migration, and death, the number of persons tested for HIV decreased over-time [26]. This analysis used data from the 2018 sero-survey round in this population. Data from the sero-survey were individually-linked to the Siaya HDSS database to extract data on date of birth, sex, marital status, and village of residence [24]. S1 Table shows differences in participants date of birth recorded in the Siaya HDSS database and HIV test results database.

### ANC data

ANC clinics included in the study represented all government owned health facilities (7 health centres and 6 dispensaries) located within Gem region of Siaya HDSS. All of these health facilities are primarily staffed by clinical officers and nurses, both of whom provide ANC services to pregnant women [27]. Compared with dispensaries, health centres provide a wider range of health services that includes vaginal deliveries.

Details of the ANC services provided for all pregnant women were logged into the ANC register and digitized for research purposes. This included attributes of the ANC client and her pregnancy (parity, gravidity, gestation, and village of residence) and laboratory test (syphilis and HIV test results). Point-of-contact interactive record linkage (PIRL) [28] was used to link ANC clients’ identifiers to their HDSS records. To that end, a fieldworker stationed in the waiting area of the ANC clinic conducted a probabilistic search of the HDSS database and confirmed the match with the ANC clinic attendee.

Our analysis included the subset of the ANC population with matched HDSS records to ensure that the ANC population corresponded to the same geographic area as the general population.

### HIV testing procedure for ANC and general population

The adult testing algorithm for prevalent HIV infection used in 2018 in Kenya was the third generation *Alere Determine*^*TM*^ *HIV-1/2* (Alere Medical Co. Ltd, Chiba, Japan), followed by third generation *First Response HIV 1-2*.*O*^™^ (Premier Medical Corporation Ltd., Kachigam, India), with DNA polymerase chain reaction (PCR) used to settle any discrepant results. Women in the ANC and general population with documented HIV positive status were not re-tested and the year of their HIV positive test was recorded.

### Data analysis

Data analysis was performed using Stata 15.1 (College Station, Texas, USA). We report descriptive statistics for both data sources and compute total and age-specific HIV prevalence by dividing the total number of HIV-positive individuals by the total number tested in that age group. P-values were obtained from the chi-square test. Because HIV prevalence varies by age, any differences in the total HIV prevalence between two populations could be due to differences in population age distribution and/or age-specific HIV prevalence rates. Kitagawa’s method [29] was used to decompose the difference in total HIV prevalence between ANC population *Y*_*A*_ and the general population *Y*_*S*_ into a structural component depending on the difference in the age distribution of these populations (*x*_*A*,*i*_ − *x*_*S*,*i*_) and a second component accounting for the difference in the age-specific prevalence (y_*A*,*i*_ − y_*S*,*i*_), as shown in Eq 1. A positive value for these terms indicates that this component increases the ANC prevalence relative to the general population and vice versa.


$$Y_{A}-Y_{s}=\sum_{i=15,5}^{45}\left(x_{A,i}-x_{S,i}\right)\cdot {\text{y}}_{A,i}+\sum_{i=15,5}^{45}\;x_{S,i}\cdot \left({\text{y}}_{A,i}-{\text{y}}_{S,i}\right)$$ (1)

### Ethics statement

Ethical approval for the study was obtained from the Kenya Medical Research Institute Scientific Ethics Review Unit (Ref No. 1801 and 3589) and the institutional review board of the London School of Hygiene and Tropical Medicine (Ref No. 14458). Written parental consent and individual assent were obtained for those aged 15–17 years; and written individual consent was obtained from adults and emancipated minors (such as pregnant, parous, or married girls aged 15–17 years) before study participation. All analyses were performed on anonymized data.

## Results

### Population characteristics

#### ANC population

Table 1 summarizes the demographic characteristics of the ANC dataset and general population sample. The ANC population consisted of 1,754 Gem residents who visited 13 ANC clinics between February 2018 and November 2018. Of these, 29 pregnant women declined to participate in the study. The number of pregnant women enrolled per clinic ranged from 27 to 286. All but one of the enrolled participants had valid HIV test results. Thus, 1,724 HIV tested women were included in the analysis.

**Table 1:** Sociodemographic characteristics of ANC and general population.

	ANC population	General population
**Total**	1,724	2,019
**Age in years (IQR)**	24 (20–29)	33 (23–41)
Missing	0	0
**Marital status**		
Married	1,324 (76.8%)	1,173 (59.1%)
Single	355 (20.6%)	548 (27.7%)
Divorced/Separated	22 (1.3%)	42 (2.1%)
Widowed	22 (1.3%)	221 (11.1%)
Missing	1	35
**Parity (IQR)**	1 (0–3)	-
Missing	13	-
**Trimester of pregnancy**		
First	271 (15.9%)	-
Second	788 (46.2%)	-
Third	645 (37.9%)	-
Missing	20	-
**Residency in the HDSS area**		
0–3 months	283 (16.4%)	29 (1.4%)
4–12 months	248 (14.4%)	47 (2.3%)
13+ months	1,193 (69.2%)	1,943 (96.3%)
Missing	0	0

The median age of the women attending ANC was 24 years (IQR: 20–29 years). The median number of reported live births prior to the current pregnancy was one (IQR: 0–3) and 27.5% were nulliparous at the time of enrolment. More than three quarters of the women (76.8%) were married, one fifth (20.6%) were unmarried, and less than 3% were widowed, divorced, or separated. Overall, 20.1% had their first clinic visit during the first trimester of their pregnancy and 57.5% had their first ANC visit during the second trimester. Seventy percent of the women had lived in the HDSS area for more than a year. The median distance travelled to the ANC clinic was 1.2 km.

#### General population

A total of 2,028 women aged 15–49 years residing in Gem (Siaya HDSS) were approached to participate in the 2018 sero-survey. Of these, three declined HIV testing and six had an indeterminate HIV test result. Thus, 2,019 women who had valid HIV test results were included in the analysis. The number of women who participated in the 2018 sero-survey per village of residency ranged from 1 to 48. The median age of the women in this sample was 33 years (IQR: 23–41 years). Of these women, 59.1% were married, 27.7% were single, 11.1% were widowed and 2.1% were separated or divorced from their partners. The majority (96.3%) had lived in the HDSS for more than one year. Nearly all women (99.8%) had previously participated in a sero-survey conducted by the HDSS team. Half of the women were living within 2.1 km and 75% within 2.9 km of a health facility providing ANC and HIV testing services in the HDSS.

### Age structure of the ANC and general populations

**Fig 1:**
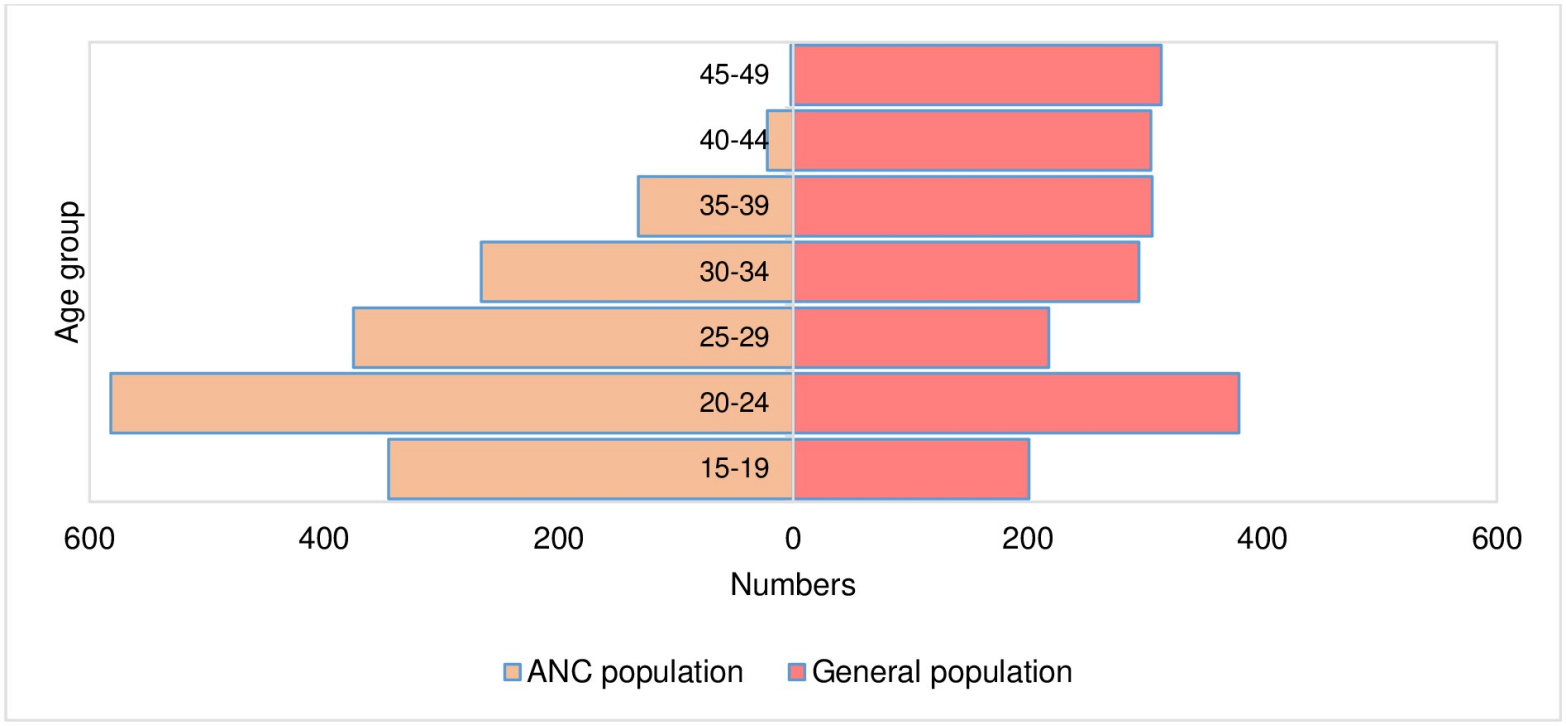
Population pyramid of ANC and general population, 2018. (In absolute numbers).

Fig 1 shows the age distribution of the ANC population and the general population sample (by five-year age groups). The ANC population was much younger than the women who participated in the sero-survey. One fifth of the ANC population were aged between 15 and 19 years compared with one tenth of the general population sample. Conversely, one third (30.6%) of the women in the general population were in the 40-49-year age group compared with 1.4% of the ANC population.

### HIV prevalence

Overall HIV prevalence in the ANC population was 18.0% (N = 1,724; 95% CI 16.3–19.9%) compared with 18.4% (N = 2,019; 95% CI 16.8–20.2%) in the general population sample. Overall, 65.1% of the ANC women had their ANC clinic records matched to an HDSS records. The HIV prevalence among ANC clients who were matched to an HDSS record was 19.7% (N = 1,122; 95% CI 17.5–22.1%), compared with 15.0% (N = 602; 95% CI 12.3–18.0%) among those who were not matched to an HDSS record.

#### HIV prevalence by selected sociodemographic background characteristics

Table 2 shows HIV prevalence in both data sources by background characteristics. In both data sources, HIV prevalence was highest among widows (77.3% in the ANC data (N = 22) and 55.7% in the general population sample (N = 221)), followed by married women (ANC: 19.6% (N = 1,324); general population: 18.3% (N = 1,173)) and never married women (ANC: 7.0% (N = 355); general population: 4.2% (N = 548)). In both populations, over 97% of women living with HIV were taking ART or were initiated onto ART at their ANC visit in 2018.

**Table 2:** Sociodemographic background characteristics of study participants, by HIV status and study population.

	ANC population (N = 1,724)				General population (N = 2,019)			
	HIV prevalence (%)	HIV negative	HIV positive	χ^2^ p-value	HIV prevalence (%)	HIV negative	HIV positive	χ^2^ p-value
**Total**	18.0	1,413	311		18.4	1,647	372	
**Marital status**				<0.001				<0.001
Married	19.6	1065	259		18.3	958	215	
Single	7.0	330	25		4.2	525	23	
Divorced/Separated	40.9	13	9		14.3	36	6	
Widowed	77.3	5	17		55.7	98	123	
Missing	100.0	0	1		14.3	30	5	
**Parity (IQR)**		1 (0–3)	3 (1–4)			-	-	
Missing		13	7			-	-	
**Time resident in HDSS area**				0.001				0.003
0–3 months	11.3	251	32		37.9	18	11	
4–12 months	15.7	209	39		6.4	44	3	
13+ months	20.1	953	240		18.4	1,585	358	
Missing	0	0	0		0	0	0	
**Matched to HDSS records**				0.015				
No	15.0	512	90			-	-	
Yes	19.7	901	221			-	-	
Missing	0	0	0			-	-	
**On ART** (*column %*)								
No			2				9	
Yes			301				343	
Missing			8				20	

Data are presented as median (IQR) for continuous measures, with % of the total population which are positive provided for each group within categorical measures in the “Positive” column, with the exception for the “On ART” variable which is only provide for the positive group.

^In general population sample, ART uptake was self-reported. In ANC population, data were extracted from self-report (n = 30) and comprehensive care centre (CCC) records (n = 271).

In the ANC population, HIV prevalence was lower among women who had lived in the HDSS area for less than 12 months compared to those who had lived in the HDSS for over one year (p-value = 0.001).

### Age-specific HIV prevalence

#### Comparison of age-specific HIV prevalence in the ANC and general population

Table 3 reports age-specific HIV prevalence estimates in the ANC and general population. In the ANC population, age-specific prevalence was higher than in the general population sample at every age, but confidence intervals sometimes overlapped. In the ANC population, HIV prevalence increased with age without any reversal at older ages, but the number of pregnant women above age 40 was small and the uncertainty around these estimates was large. In the general population sample, HIV prevalence increased with age and peaked in the age group 30–35 at 33.0% (95% CI 27.9–38.5%). Point estimates of HIV prevalence were lower at older ages, but confidence intervals were wide.

**Table 3:** Age-specific HIV prevalence estimates, by study population.

	ANC population	General population	ANC population matched to HDSS records
	HIV prevalence % (95% CI)	HIV prevalence % (95% CI)	HIV prevalence % (95% CI)
**Total HIV prevalence**	311/1,724	372/2,019	221/1,122
	18.0 (16.3–19.9)	18.4 (16.8–20.2)	19.7 (17.5–22.1)
**Age group**			
**15–19**	14/345	8/201	8/195
	4.1 (2.4–6.8)	4.0 (2.0–7.8)	4.1 (2.1–8.0)
**20–24**	72/582	16/380	40/333
	12.4 (10.0–15.3)	4.2 (2.6–6.8)	12.0 (8.9–16.0)
**25–29**	79/375	24/218	56/266
	21.1 (17.2–25.5)	11.0 (7.5–15.9)	21.1 (16.5–26.4)
**30–34**	86/266	71/295	64/198
	32.3 (26.9–38.2)	24.1 (19.5–29.3)	32.3 (26.1–39.2)
**35–39**	48/132	101/306	43/109
	36.4 (28.5–45.0)	33.0 (27.9–38.5)	39.4 (30.6–49.0)
**40–44**	11/22	79/305	10/20
	50.0 (28.7–71.3)	25.9 (21.3–31.1)	50.0 (27.7–72.3)
**45–49**	1/2	73/314	0/1
	50.0 (0.0–100.0)	23.2 (18.9–28.3)	0.0 (0.0–0.0)

The age-specific HIV prevalence among the sub-group of the ANC population who were matched to an HDSS record was similar to the entire group of women who visited ANC clinics.

### Decomposition of the difference in the total HIV prevalence in the ANC and the general population of women at reproductive ages

**Fig 2:**
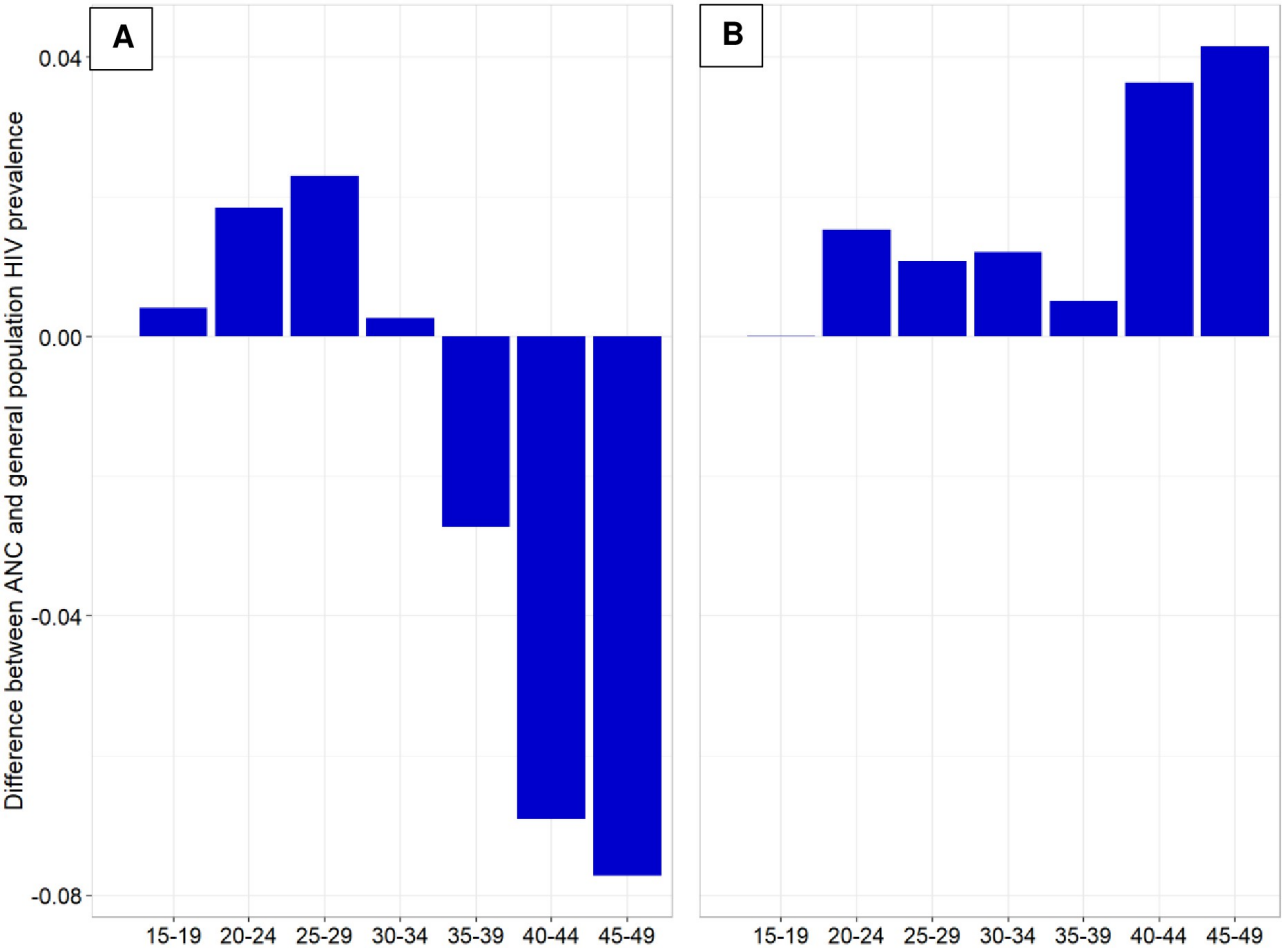
Age group contributions to the difference in HIV prevalence between the ANC and general population, decomposed into a component attributable to a difference in the age structure (panel A) and a component attributable to differences in age-specific HIV prevalence (panel B). Panel A. Contribution of age-compositional differences. Panel B. Contribution of differences in age-specific HIV prevalence.

Fig 2 illustrates the age group contribution to the difference in HIV prevalence between the ANC and general population. The decomposition differentiates the prevalence difference that is due to differences in the age structure between the two populations (the first term in Eq (1) and shown in panel A), and the portion that is attributable to differences in age-specific HIV prevalence (the second term in Eq (1) and shown in panel B). As also shown in Table 3, HIV prevalence in the ANC population is higher at every age. This increases the overall HIV prevalence in the ANC population relative to the general population (panel B). The sum of these age-specific prevalence differences would increase the ANC prevalence by 12.15 percentage points if the age distribution of ANC women was the same as the general population age distribution. Conversely, the younger age structure of the ANC population decreases the total ANC prevalence versus the general population prevalence because HIV prevalence was lower among younger women, who are disproportionately represented in the ANC population (panel A). Summed over all age groups, this reduces the ANC prevalence by 12.53 percentage points. The total effects of the population composition (panel A) and the difference in rates (panel B) nearly cancel each other out, explaining why the observed HIV prevalence in both populations is nearly identical.

## Discussion

In this study in Siaya County (Western Kenya), HIV prevalence among ANC attendees (18.0%; 95% CI 16.3–19.9%) was similar to that amongst women of reproductive age in a general population sample (18.4%; 95% CI 16.8–20.2%). This apparent correspondence in HIV prevalence in both data sources, however, concealed important disparities in the age distribution and age-specific HIV prevalence in the two populations. On the one hand, women visiting ANC were younger than women of reproductive age in the general population sample, and, because HIV prevalence is typically lower at younger ages, this tends to reduce the total prevalence in the ANC population compared to the general population. Age-specific HIV prevalence was, on the other hand, higher in the ANC population at every age and this elevates the total prevalence estimate for the ANC population relative to the general population. These two effects cancelled each other out, and it is therefore coincidental that the overall HIV prevalence estimates derived from the ANC data and the general population sample were so well aligned. Restricting the ANC population to women who were matched to an HDSS record did not alter this comparison. It is also notable that HIV prevalence estimates from both data sources were equivalent among the youngest women [15–19], whereas studies conducted earlier in the HIV epidemic often reported higher prevalence among pregnant young women attending ANC because this source tended to select young women who are sexually active [30]. This may be because historically adolescent women living with HIV were most likely recently infected with HIV, whereas now a large proportion are likely to be long-term survivors of perinatal HIV infection, which does not select for sexually active women.

The comparison of (age-specific) HIV prevalence in the two date sources indicates that extrapolation of programmatic ANC-based HIV prevalence estimates to the general population has to be made with caution. In the pre-ART era, it was common practice to adjust ANC-based prevalence estimates downward by multiplying by a factor of 0.8 to approximate general population prevalence in women of reproductive age [11]. These data suggest that such a practice may no longer be justifiable, and our study lends support to the assertion that the relationship between ANC prevalence and general population prevalence is dynamic and not fully captured by a single adjustment factor [12]. It also underscores that the difference between both data sources cannot be resolved by mere age-standardisation. To the contrary, the results from this study suggest that this would amplify the differences.

There are several plausible reasons why the relationship between ANC and general population HIV prevalence estimates may be altered by the expansion of ART and the integration of prevention of mother-to-child transmission (PMTCT) of HIV and ANC services. First, the expansion of treatment is likely to have reduced the gap in fertility between women living with HIV and HIV negative women [31, 32], and increased HIV prevalence in (older) pregnant women [33–35]. Second, data from the same study site suggest that HIV-positive pregnant women were more frequent users of ANC services than HIV negative women, including earlier first ANC visits and shorter visit intervals [36]. More intense ANC use by women living with HIV may be prompted by the need for close clinical monitoring and access to PMTCT services. This phenomenon may lead to the over-representation of women living with HIV in ANC surveillance data, although the overall coverage of ANC services in this setting is high [37].

The expansion of ART and increased survival of women living with HIV may produce a third, albeit temporary, over-representation of women living with HIV in the ANC dataset in the older age groups. A cohort of women living with HIV with low fertility at younger ages may have recuperated some of the forgone births once ART became available [31, 32, 38]. This phenomenon could contribute to an overrepresentation of women living with HIV in the ANC data since their negative counterparts have already finished childbearing. If so, this effect is likely to be transient as the widespread availability of ART means there is now no reason for younger woman living with HIV to delay childbearing. If this is the case, we expect the discrepancy between ANC and general population estimates to diminish as the cohort of women whose peak childbearing years were prior to the ART rollout complete their childbearing years. Women born in the 1970s and the first half of the 1980s would have been in their late 20s or 30s by the time they had access to ART, whereas women born since 1985 have essentially had access for their entire adult lives. If this hypothesis is correct, policymakers can plan for a short-term increased need for ANC services among pregnant women living with HIV, who will attend a greater number of visits and result in increased healthcare utilization.

### Study strengths and limitations

This study provides timely new evidence comparing HIV prevalence estimates between ANC attendees and the general population, since the expansion of treatment eligibility among pregnant women through Option B+ and UTT. A key strength of this study is the inclusion of population-based data collected from ANC attendees, as well as the general population, residing in the same region. However, the small number of ANC attendees at older ages (40–49 year) affects the precision of HIV prevalence estimates in this age group. Another limitation was that women in the general population aged between 25 and 29 years were less likely to be found at home when the home-based HIV testing exercise was being conducted. Therefore, non-participation could affect interpretation of our results as about a tenth of the women in this age-group were not tested during the 2018 HIV sero-survey.

## Conclusion

HIV prevalence among ANC attendees in Siaya County (Western Kenya) in 2018 was largely similar to HIV prevalence in a general population sample of women of reproductive age, but this correspondence conceals important differences in the age distribution of these populations as well as their age-specific HIV prevalence. Extrapolation of programmatic ANC data to the general population should be done with caution because both the fertility of women living with HIV as well as the intensity of services use is likely to change in response to the availability of treatment and how these are integrated in ANC services provision.

## Supporting information

S1 Table Comparison of date of birth in HIV test results database and Siaya Health Demographic Surveillance System database. *Differences between date of birth in HIV test results database and Siaya HDSS database.
(DOCX)
