# Supplementary material for: Comparison of programmatic data from antenatal clinics with population-based HIV prevalence estimates in the era of universal test and treat in western Kenya
Source: PLoS One. 2023 Jun 26;18(6):e0287626. doi: 10.1371/journal.pone.0287626 (PMC10292704; doi:10.1371/journal.pone.0287626)
Supplement: S1 Table — *Differences between date of birth in HIV test results database and Siaya HDSS database. (DOCX) [file pone.0287626.s001.docx]

**S1 Table.**

|  | **Age category in Siaya HDSS database** | | | | | | |  |
| --- | --- | --- | --- | --- | --- | --- | --- | --- |
| **Age difference***  **in years** | **15-19** | **20-24** | **25-29** | **30-34** | **35-39** | **40-44** | **45-49** | **Total** |
|  | **n (%)** | **n (%)** | **n (%)** | **n (%)** | **n (%)** | **n (%)** | **n (%)** | **n (%)** |
| -10 + | 1 (0.5) | 3 (0.8) | 0 (0.0) | 1 (0.3) | 0 (0.0) | 0 (0.0) | 2 (0.6) | 7 (0.3) |
| -10 - -5 | 1 (0.5) | 0 (0.0) | 1 (0.5) | 1 (0.3) | 1 (0.3) | 2 (0.7) | 3 (1.0) | 9 (0.4) |
| -5 - -2.5 | 1 (0.5) | 6 (1.6) | 3 (1.4) | 3 (1.0) | 5 (1.6) | 4 (1.3) | 10 (3.2) | 32 (1.6) |
| -2.5 - -1 | 20 (10.0) | 46 (12.1) | 16 (7.3) | 29  (9.8) | 38 (12.4) | 38 (12.5) | 32 (10.2) | 219 (10.8) |
| 0 - 0 | 157 (78.1) | 245 (64.5) | 153 (70.2) | 216 (73.2) | 216 (70.6) | 224 (73.4) | 240 (76.4) | 1,451 (71.9) |
| 1 - 2.5 | 19 (9.5) | 56 (14.7) | 21 (9.6) | 15  (5.1) | 22 (7.2) | 15 (4.9) | 7  (2.2) | 155 (7.7) |
| 2.5 - 5 | 1  (0.5) | 22 (5.8) | 18 (8.3) | 20  (6.8) | 16 (5.2) | 12 (3.9) | 11  (3.5) | 100 (5.0) |
| 5 - 10 | 1 (0.5) | 2 (0.5) | 6 (2.8) | 10 (3.4) | 7 (2.3) | 7 (2.3) | 7 (2.2) | 40 (2.0) |
| 10+ | 0 (0.0) | 0 (0.0) | 0 (0.0) | 0 (0.0) | 1 (0.3) | 3 (1.0) | 2 (0.6) | 6 (0.3) |
| **Total** | **201** | **380** | **218** | **295** | **306** | **305** | **314** | **2,019** |
